# Fetuin-A Attenuates HMGB1/TLR4-Mediated Ovarian Ischemia/Reperfusion Injury

**DOI:** 10.3390/biomedicines14102282

**Published:** 2026-10-08

**Authors:** Ezgi Tolu Cenk, Selim Afşar, Özgür Bulmuş, Figen Efe Çamili, Mustafa Hilmi Yaranoğlu, Mine İslimye Taşkın, Ayla Solmaz Avcıkurt, Gürhan Güney, Merve Akış Yılmaz, Gülay Turan, Özge Özmen, Akın Usta, Ceyda Sancaklı Usta

**Affiliations:** 1 Department of Obstetrics & Gynecology, Balıkesir City Hospital, Balıkesir 10100, Turkey; 2 Department of Obstetrics & Gynecology, Faculty of Medicine, Balıkesir University, Balıkesir 10145, Turkey; selim.afsar@balikesir.edu.tr (S.A.); figen.camili@balikesir.edu.tr (F.E.Ç.); minetaskin@balikesir.edu.tr (M.İ.T.); gurhanguney@yahoo.com (G.G.);; 3 Department of Physiology, Faculty of Medicine, Balıkesir University, Balıkesir 10145, Turkey; ozgur.bulmus@balikesir.edu.tr; 4 Experimental Animals Production, Care, Application and Research Center, Balıkesir University, Balıkesir 10145, Turkey; mhyaranoglu@balikesir.edu.tr; 5 Department of Medical Biology, Faculty of Medicine, Balıkesir University, Balıkesir 10145, Turkey; aylaavcikurt@hotmail.com; 6 Department of Biochemistry, Faculty of Medicine, Balıkesir University, Balıkesir 10145, Turkey; merve.akis@balikesir.edu.tr; 7 Department of Pathology, Faculty of Medicine, Balıkesir University, Balıkesir 10145, Turkey; gulaytr@yahoo.com.tr; 8 Department of Pathology, Hatay Training and Research Hospital, Hatay 31001, Turkey

**Keywords:** ovarian torsion, ischemia/reperfusion injury, Fetuin-A, HMGB1, TLR4, anti-Müllerian hormone (AMH), apoptosis, autophagy, oxidative stress

## Abstract

**Background/Objectives:** Adnexal torsion causes ovarian ischemia/reperfusion injury and may impair ovarian reserve. This study evaluated the protective effects of Fetuin-A in a rat torsion/detorsion model. **Methods:** Twenty-eight female Wistar rats were assigned to Sham, T/D, T/D+Fetuin-A treatment, and Fetuin-A pretreatment+T/D groups. Bilateral ovarian torsion was induced for 3 h followed by 2 h of detorsion/reperfusion, and Fetuin-A was administered intraperitoneally at 100 mg/kg. Serum cytokines and AMH, qRT-PCR markers, histopathological injury, and immunohistochemical expression of Fetuin-A, HIF-1α, GPx, and Bcl-2 were evaluated. **Results:** T/D induced a systemic inflammatory response, reduced AMH, increased HMGB1/TLR4-associated and cell-stress markers, and caused marked ovarian histopathological injury. Fetuin-A attenuated inflammatory and molecular stress responses, while pretreatment produced the most consistent protection, preserving AMH, reducing histopathological injury and HIF-1α immunoreactivity, and restoring GPx, Bcl-2, and Fetuin-A immunoreactivity. Exploratory STRING analysis identified functional enrichment related to oxidative stress, autophagy, apoptosis, and HIF-1 signaling, providing hypothesis-generating context without establishing causal pathway activity. **Conclusions:** These findings indicate that Fetuin-A administration, particularly before torsion, was associated with reduced inflammatory and tissue-stress responses during early ovarian ischemia/reperfusion injury. Further studies are needed to determine optimal dosing, clinically feasible timing, and long-term reproductive outcomes.

## 1. Introduction

Adnexal torsion is a gynecological emergency defined by partial or complete rotation of the ovary and fallopian tube around their vascular axis [1]. Although its true population incidence remains uncertain, adnexal torsion accounts for approximately 2.7% of gynecological surgical emergencies and occurs most frequently during the reproductive years [1]. This mechanical obstruction compromises ovarian blood flow and precipitates acute ischemia. Although prompt surgical detorsion remains the primary therapeutic intervention, restoration of blood flow can trigger ischemia/reperfusion injury [2,3,4]. During reperfusion, sudden tissue reoxygenation generates reactive oxygen species, damage-associated molecular patterns, and proinflammatory cytokines, ultimately contributing to follicular atresia and decline in ovarian reserve [3,5,6].

At the molecular level, ovarian ischemia/reperfusion injury is driven in part by activation of damage-associated molecular signaling, including the HMGB1/TLR4 axis [7,8,9]. In response to cellular stress, extracellular HMGB1 can act as a ligand for TLR4, promoting sterile inflammation and downstream production of TNF-α, IL-1β, and IL-6 [7,8]. Ischemia/reperfusion may also alter autophagy-related signaling, which can be explored through markers such as Beclin-1 and MAP1LC3; however, changes in these markers require cautious interpretation because they do not independently establish autophagic flux [10,11]. The convergence of inflammatory, autophagy-related, and apoptosis-associated responses may contribute to ovarian tissue injury after adnexal torsion [3,4].

Fetuin-A (α2-HS glycoprotein; AHSG) is a predominantly liver-derived circulating glycoprotein with context-dependent immunometabolic and cytoprotective effects. Its reported molecular relationships include TLR4-dependent lipid-induced inflammatory signaling [12] and hypoxia-responsive regulation involving HIF signaling [13,14]. Fetuin-A has also been linked to modulation of HMGB1-associated sterile inflammatory responses [15,16] and oxidative-stress and cell-death pathways [17,18]. These actions appear to depend on the underlying stimulus, tissue, exposure pattern, and disease setting: chronic elevation of Fetuin-A has been associated with adverse metabolic signaling, whereas exogenous Fetuin-A has limited inflammation and tissue injury in experimental systemic inflammation, cerebral ischemia, traumatic brain injury, intestinal ischemia/reperfusion, and hypoxic stress models [14,15,16,17,18,19]. Despite these experimental effects, Fetuin-A is not an established therapeutic agent in clinical practice and, in human studies, has mainly been investigated as a circulating biomarker rather than as an administered treatment. Its role in ovarian ischemia/reperfusion injury remains unknown. Therefore, whether exogenous Fetuin-A can attenuate HMGB1/TLR4-associated inflammation and preserve ovarian tissue and the AMH-related ovarian reserve profile during adnexal torsion requires experimental evaluation.

In the present study, we evaluated whether intraperitoneal Fetuin-A attenuates ovarian ischemia/reperfusion injury after adnexal torsion in a standardized rat model. Specifically, we examined its effects on HMGB1/TLR4-associated inflammatory signaling, systemic cytokine activity, AMH as an exploratory marker of ovarian reserve, autophagy-related and apoptosis-associated molecular activation, histopathological injury, and immunohistochemical indicators of hypoxia-related stress, antioxidant defense, and anti-apoptotic response. We hypothesized that Fetuin-A administration, particularly before torsion, would attenuate HMGB1/TLR4-associated inflammatory signaling, reduce ovarian tissue injury, and preserve AMH compared with untreated torsion/detorsion.

## 2. Results

### 2.1. Serum Biochemical Findings and Ovarian Reserve

**Figure 1:**
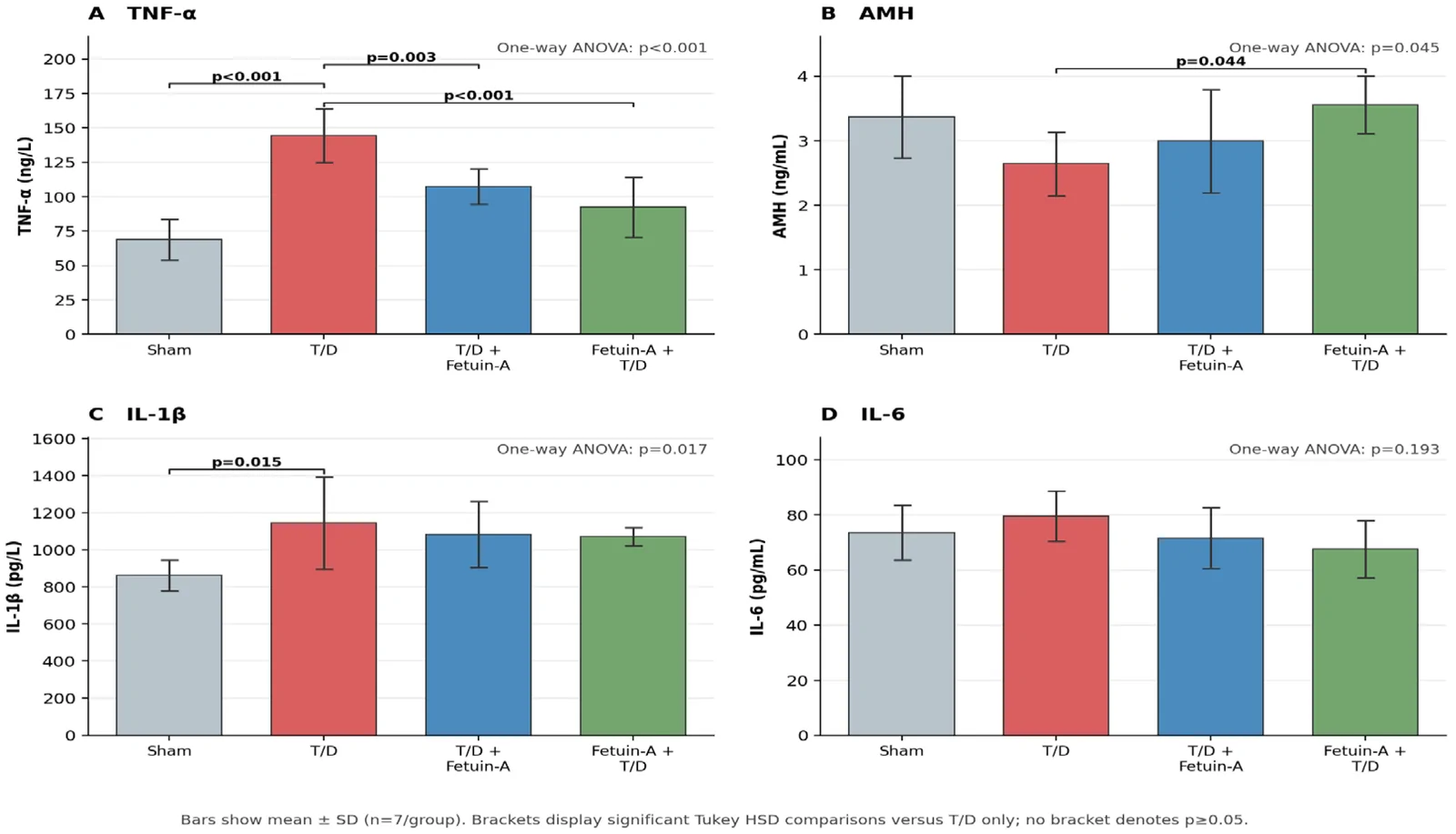
Serum IL-1β, IL-6, TNF-α, and AMH levels across the experimental groups. Overall intergroup differences were observed for IL-1β, TNF-α, and AMH, but not IL-6. Compared with untreated T/D animals, Fetuin-A treatment reduced TNF-α (Tukey-adjusted *p* = 0.0032), whereas Fetuin-A pretreatment reduced TNF-α (Tukey-adjusted *p* < 0.001) and preserved AMH (Tukey-adjusted *p* = 0.0445). Data are presented as mean ± SD (*n* = 7 per group). Significance annotations indicate Tukey-adjusted comparisons with the untreated T/D group.

As shown in Figure 1, serum biomarkers were compared among the Sham group, which underwent ovarian exposure and manipulation without torsion, the untreated torsion/detorsion (T/D) group, the Fetuin-A treatment group, and the Fetuin-A pretreatment group. Compared with untreated T/D animals, Fetuin-A treatment reduced TNF-α (Tukey-adjusted *p* = 0.0032), while pretreatment reduced TNF-α (*p* < 0.001) and preserved AMH (*p* = 0.0445). No significant treatment effects were detected for IL-6. Overall, Fetuin-A—particularly when administered before torsion—attenuated TNF-α activity and helped preserve the AMH-related ovarian reserve profile.

### 2.2. Quantitative mRNA Expression Analysis

**Figure 2:**
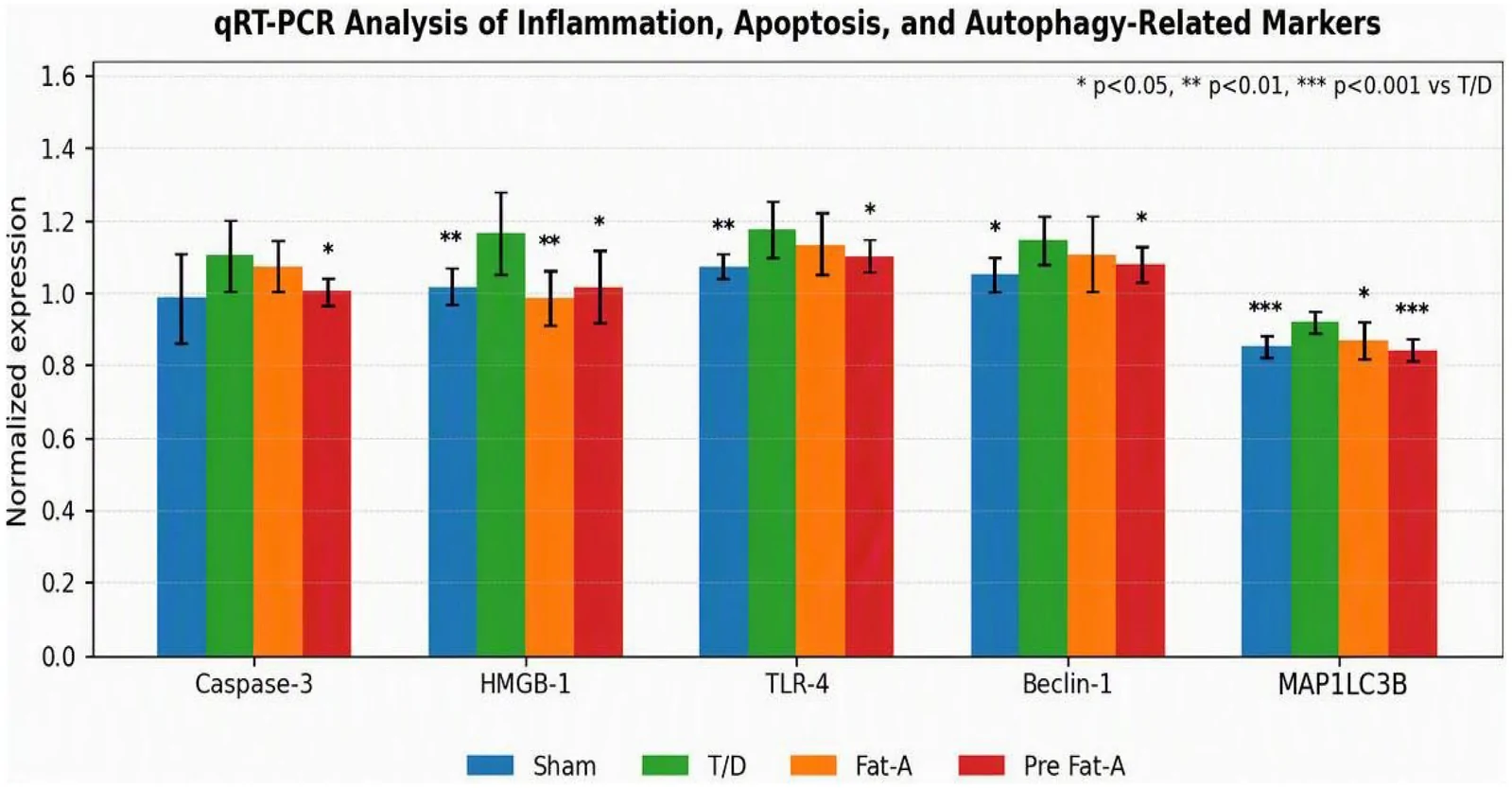
qRT-PCR expression of inflammatory, apoptosis-associated, and autophagy-related markers across the experimental groups. Relative mRNA expression of *Casp3*, *Hmgb1*, *Tlr4*, *Becn1*, and *Map1lc3b* was assessed. Fetuin-A pretreatment produced the most consistent attenuation of *Hmgb1*/*Tlr4*-associated inflammatory signaling and *Map1lc3b* mRNA expression; nominal pairwise findings for *Casp3* and *Becn1* were not interpreted because their omnibus tests were non-significant. Data are presented as mean ± SD (*n* = 7 per group); * *p* < 0.05; ** *p* < 0.01; *** *p* < 0.001.

As shown in Figure 2, qRT-PCR analysis demonstrated significant intergroup differences in *Hmgb1* mRNA (*p* = 0.0035), *Tlr4* mRNA (*p* = 0.0296), and *Map1lc3b* mRNA (*p* = 0.0022), whereas *Casp3* and *Becn1* mRNA did not differ significantly in the overall ANOVA. T/D increased *Map1lc3b* mRNA expression, whereas *Becn1* mRNA was numerically increased but did not differ significantly in the overall analysis. Compared with untreated T/D animals, Fetuin-A treatment reduced *Hmgb1* (*p* = 0.0044) and *Map1lc3b* (*p* = 0.0386) mRNA expression, whereas Fetuin-A pretreatment reduced *Hmgb1* (*p* = 0.0229), *Tlr4* (*p* = 0.0415), and *Map1lc3b* (*p* = 0.0003) mRNA expression. Because the omnibus tests for *Casp3* and *Becn1* were non-significant, their nominal pairwise findings were not interpreted as evidence of treatment effects. These findings indicate that Fetuin-A pretreatment produced the most consistent attenuation of *Hmgb1*/*Tlr4*-associated inflammatory signaling and *Map1lc3b* mRNA expression.

### 2.3. STRING Functional Association Analysis

Exploratory STRING analysis of the selected nine-protein panel in *Rattus norvegicus* identified 17 high-confidence functional associations, compared with 1 expected association (PPI enrichment *p* = 5.11 × 10^−15^). The network had an average node degree of 3.78 and an average local clustering coefficient of 0.704. Eight proteins formed a connected component, whereas AHSG remained disconnected at the 0.700 confidence threshold (Figure S6). Functional enrichment identified response to oxidative stress (FDR = 4.28 × 10^−5^), autophagy–animal (FDR = 4.40 × 10^−4^), apoptosis–multiple species (FDR = 2.40 × 10^−4^), and HIF-1 signaling (FDR = 0.0023) (Table S1). These findings provide hypothesis-generating context for functional relationships among the selected markers and do not establish direct physical binding or treatment-specific pathway activity.

### 2.4. Histopathological Injury and Immunohistochemical Marker Scores

**Figure 3:**
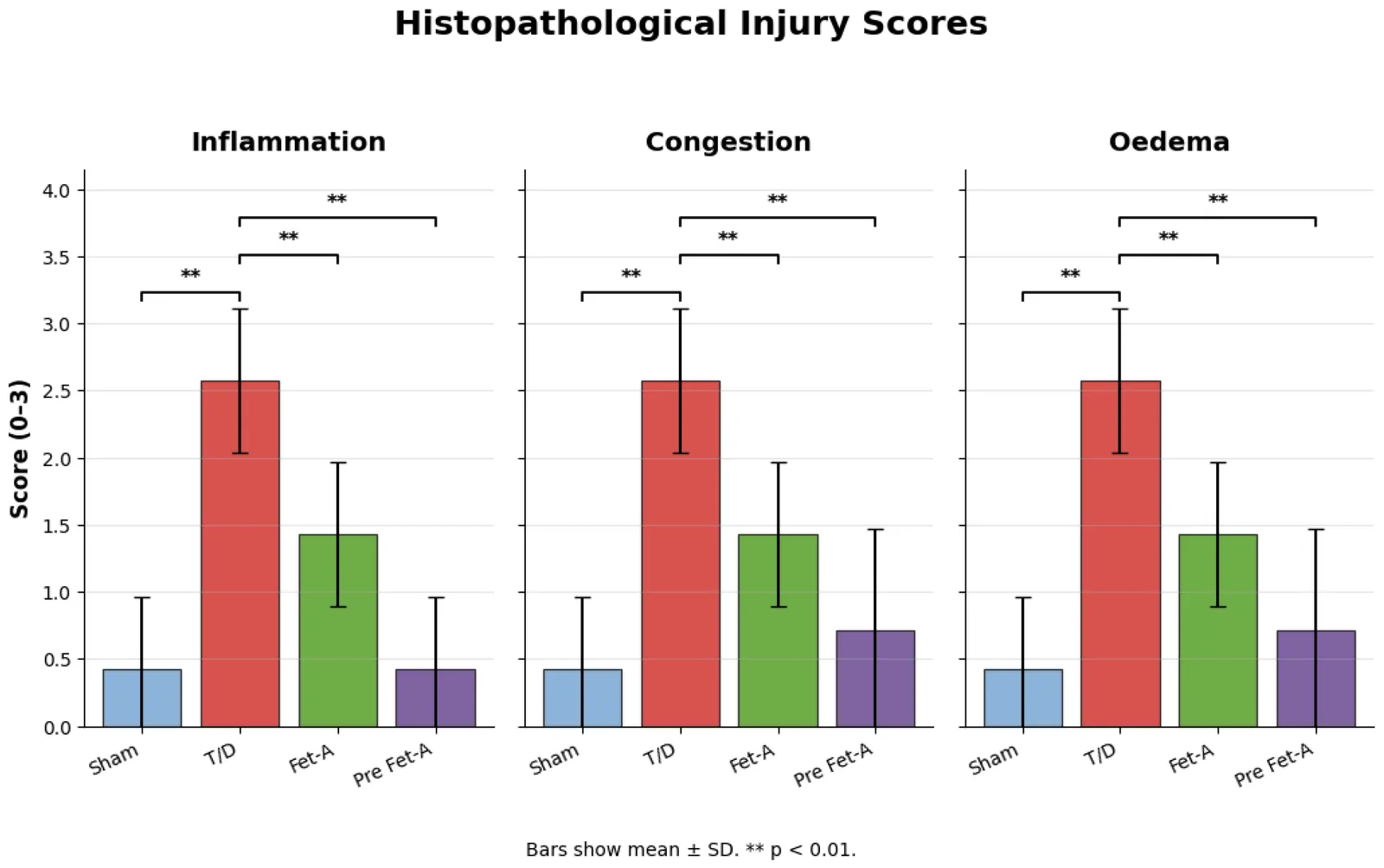
Histopathological injury scores across the experimental groups. Inflammation, congestion, and oedema scores increased after T/D and were reduced by Fetuin-A, with pretreatment showing the greatest histopathological protection. Holm-adjusted pairwise Mann–Whitney U tests versus untreated T/D showed significant differences for inflammation (Sham, *p* = 0.0046; treatment, *p* = 0.0079; pretreatment, *p* = 0.0046), congestion (Sham, *p* = 0.0046; treatment, *p* = 0.0079; pretreatment, *p* = 0.0060), and oedema (Sham, *p* = 0.0046; treatment, *p* = 0.0079; pretreatment, *p* = 0.0060). Data are presented as mean ± SD (*n* = 7 per group); ** *p* < 0.01.

As shown in Figure 3, T/D caused marked histopathological injury, with inflammation, congestion, and oedema scores increasing from 0.43 in Sham animals to 2.57 in untreated T/D animals. Representative H&E-stained sections from untreated T/D ovaries show severe inflammatory-cell infiltration in the ovarian stroma with dilated congested vessels and interstitial oedema, as well as marked vascular congestion and stromal oedema (Figure S1). Kruskal–Wallis analysis confirmed significant intergroup differences for inflammation (H = 20.085, df = 3, *p* = 0.0002), congestion (H = 18.223, df = 3, *p* = 0.0004), and oedema (H = 18.223, df = 3, *p* = 0.0004). Fetuin-A treatment reduced all three injury scores to 1.43, while Fetuin-A pretreatment showed the greatest histological protection, with inflammation returning to Sham-like levels (0.43) and lower congestion and oedema scores (0.71 each). In Holm-adjusted pairwise Mann–Whitney U tests versus untreated T/D, both Fetuin-A treatment and pretreatment reduced inflammation (adjusted *p* = 0.0079 and 0.0046, respectively), congestion (adjusted *p* = 0.0079 and 0.0060), and oedema (adjusted *p* = 0.0079 and 0.0060). Sham-versus-T/D comparisons were also significant for all three outcomes (adjusted *p* = 0.0046 for each). These findings demonstrate that Fetuin-A, particularly pretreatment, attenuated structural ovarian injury after T/D.

**Figure 4:**
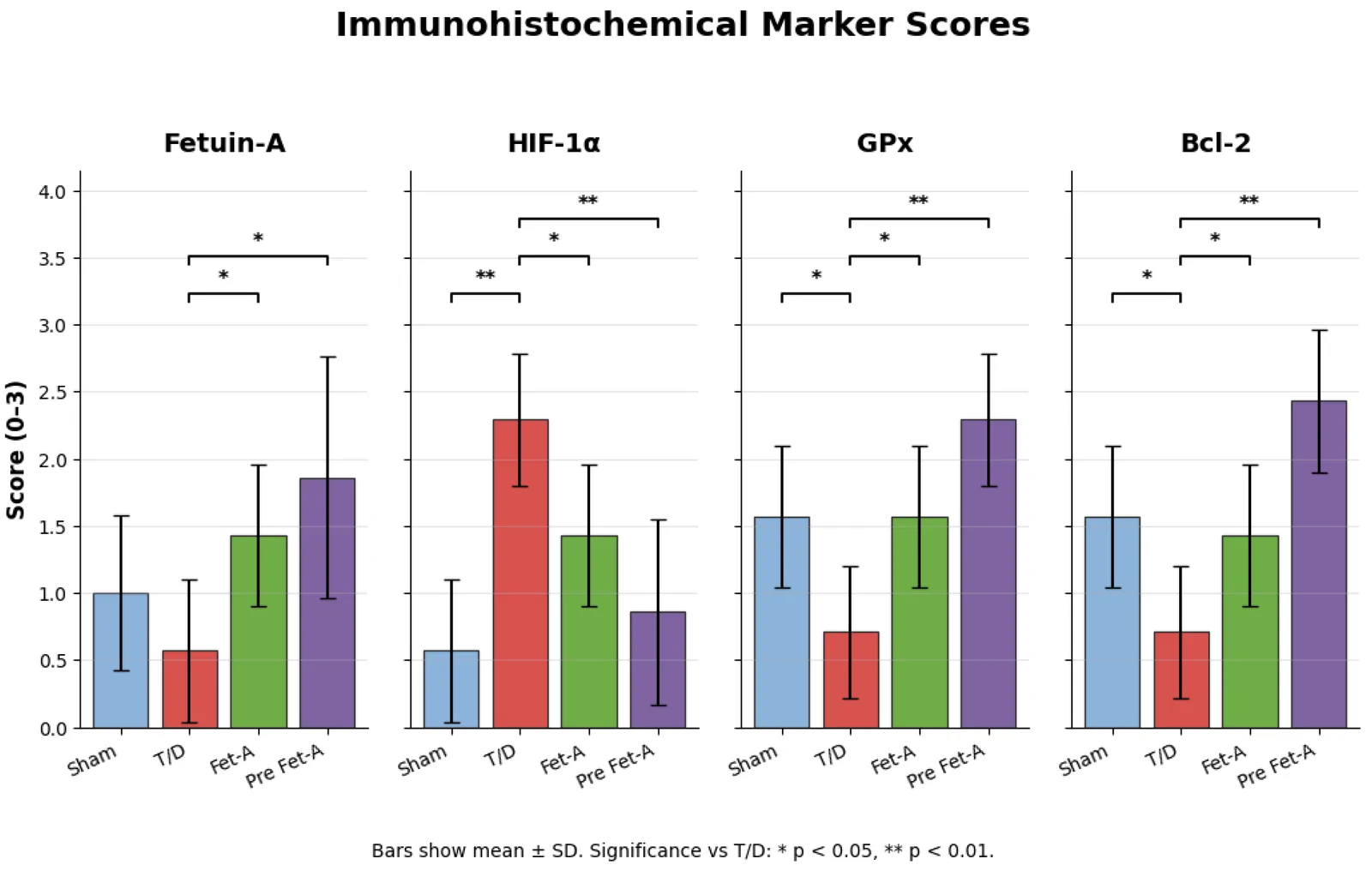
Immunoreactivity scores for Fetuin-A, HIF-1α, GPx, and Bcl-2 across the experimental groups. T/D increased HIF-1α and reduced GPx and Bcl-2 immunoreactivity, whereas Fetuin-A pretreatment increased GPx and Bcl-2 immunoreactivity and reduced HIF-1α immunoreactivity. Holm-adjusted pairwise Mann–Whitney U tests versus untreated T/D showed the following adjusted *p*-values: Fetuin-A (Sham, *p* = 0.0531; treatment, *p* = 0.0442; pretreatment, *p* = 0.0400), HIF-1α (Sham, *p* = 0.0042; treatment, *p* = 0.0178; pretreatment, *p* = 0.0074), GPx (Sham, *p* = 0.0356; treatment, *p* = 0.0356; pretreatment, *p* = 0.0039), and Bcl-2 (Sham, *p* = 0.0356; treatment, *p* = 0.0357; pretreatment, *p* = 0.0042). Data are presented as mean ± SD (*n* = 7 per group); * *p* < 0.05; ** *p* < 0.01.

Immunohistochemical findings paralleled the histopathological improvement (Figure 4). Kruskal–Wallis analysis showed significant intergroup differences in Fetuin-A (H = 10.790, df = 3, *p* = 0.0129), HIF-1α (H = 16.880, df = 3, *p* = 0.0007), GPx (H = 15.833, df = 3, *p* = 0.0012), and Bcl-2 (H = 16.564, df = 3, *p* = 0.0009) immunoreactivity scores. Compared with Sham, untreated T/D animals showed higher HIF-1α expression (0.57 to 2.29) and lower GPx and Bcl-2 scores (1.57 to 0.71 for both), indicating hypoxia-related stress with impaired antioxidant and anti-apoptotic defenses. Representative moderate HIF-1α immunoreactivity in the Fetuin-A treatment group and strong immunoreactivity in the untreated T/D group are shown in Figure S2. Fetuin-A pretreatment showed the strongest restorative pattern, with lower HIF-1α (0.86) and higher GPx (2.29) (Figure S3), Bcl-2 (2.43) (Figure S4), and Fetuin-A immunoreactivity (1.86) (Figure S5). In Holm-adjusted pairwise Mann–Whitney U tests versus untreated T/D, Fetuin-A treatment and pretreatment differed significantly for Fetuin-A immunoreactivity (adjusted *p* = 0.0442 and 0.0400, respectively), HIF-1α (adjusted *p* = 0.0178 and 0.0074), GPx (adjusted *p* = 0.0356 and 0.0039), and Bcl-2 (adjusted *p* = 0.0357 and 0.0042). Sham-versus-T/D comparisons were significant for HIF-1α (adjusted *p* = 0.0042), GPx (adjusted *p* = 0.0356), and Bcl-2 (adjusted *p* = 0.0356), but not for Fetuin-A immunoreactivity (adjusted *p* = 0.0531). These data support a tissue-protective effect of Fetuin-A through mitigation of hypoxia-related stress and restoration of antioxidant and anti-apoptotic defenses.

## 3. Discussion

The present study indicates that Fetuin-A administration is associated with coordinated protection against ovarian ischemia/reperfusion injury induced by adnexal torsion/detorsion. The protective pattern was evident across systemic, molecular, histopathological, and immunohistochemical outcomes, with pretreatment producing the most consistent benefit. Serum analysis showed that T/D enhanced inflammatory activity and reduced AMH, whereas Fetuin-A—particularly pretreatment—reduced TNF-α and helped preserve the AMH-related ovarian reserve profile [5,6]. In parallel, qRT-PCR findings showed attenuation of HMGB1/TLR4-associated inflammatory signaling [7,8,9] and reduced *Map1lc3b* mRNA expression [10,11]. These biochemical and transcriptional changes were accompanied by lower histopathological injury scores and improved immunohistochemical expression of HIF-1α, GPx, and Bcl-2 [13]. Together, these findings are consistent with an association between Fetuin-A administration and reduced inflammatory, hypoxia-related, oxidative-stress-related, and cell-stress responses after torsion/detorsion injury [2,15,17], but they do not establish direct pathway causality.

Sterile inflammation is a central component of ischemia/reperfusion injury [7]. Fetuin-A has also been identified as an endogenous ligand and adaptor for TLR4 in lipid-induced inflammatory signaling [12]. Tissue stress and cellular damage can promote release of damage-associated molecular signals such as HMGB1, which activate TLR4-dependent inflammatory pathways and amplify cytokine production [7,8,9]. In the present study, T/D increased IL-1β and TNF-α, whereas the overall IL-6 comparison was not significant. T/D also upregulated HMGB1 and TLR4 expression in ovarian tissue, supporting activation of this pathway. Fetuin-A reduced TNF-α at the serum level and suppressed HMGB1/TLR4-related molecular responses, especially when administered before torsion. This pattern is consistent with, but does not establish, an upstream modulatory effect of Fetuin-A on sterile inflammatory signaling because no pathway-specific manipulation was performed. Furthermore, in a cerebral ischemia model [16], peripheral Fetuin-A administration was shown to alleviate early ischemic damage and microglial inflammatory responses through regulation of TNF-α and IL-1β. Accordingly, the present systemic cytokine and gene-expression findings should be interpreted as associative evidence of an anti-inflammatory effect in this ovarian torsion/detorsion model rather than direct proof of pathway causality.

The immunohistochemical findings further indicate that Fetuin-A modulated hypoxia-related and cytoprotective responses. T/D increased HIF-1α expression, consistent with hypoxic stress [13], and reduced GPx and Bcl-2 expression, suggesting weakened antioxidant and anti-apoptotic defense. Fetuin-A has also been described as a hypoxia-responsive HIF target, indicating a potentially reciprocal relationship between hypoxic signaling and Fetuin-A regulation [14]. In the present model, Fetuin-A pretreatment reversed the injury-associated profile most clearly, lowering HIF-1α while increasing GPx and Bcl-2 immunoreactivity. These changes suggest that Fetuin-A-mediated suppression of inflammation is accompanied by improved redox defense and enhanced resistance to apoptosis, thereby contributing to tissue preservation after detorsion. During the detorsion period, sudden oxygen influx into the tissue leads to an increase in reactive oxygen species (ROS) and depletion of antioxidant defense mechanisms. In our study, the decrease in GPx immunoreactivity and increase in HIF-1α immunoreactivity in the T/D group were consistent with ovarian I/R models in the literature [19]. The reduction in HIF-1α and restoration of GPx immunoreactivity with Fetuin-A pretreatment are consistent with improved hypoxia-related and antioxidant responses; however, these associative findings do not establish that Fetuin-A acts as a direct antioxidant or directly regulates HIF-1α.

Histopathological findings provide structural confirmation of these molecular effects. T/D markedly increased inflammation, congestion, and oedema scores, indicating successful induction of ovarian tissue injury [3,4]. Fetuin-A reduced these injury parameters, with pretreatment producing the greatest improvement and returning inflammation scores toward Sham-like levels. The parallel reduction in histological injury and improvement in HIF-1α, GPx, and Bcl-2 expression suggests that Fetuin-A-mediated molecular protection translated into preservation of ovarian tissue architecture [12,13,14,15,17].

The qRT-PCR and immunohistochemical data also suggest that Fetuin-A influenced the balance between cell stress and survival. T/D increased *Map1lc3b* mRNA expression, whereas *Becn1* mRNA was numerically increased but did not differ significantly in the overall analysis [10,11]; T/D was also associated with reduced Bcl-2 immunoreactivity. Because the overall tests for *Casp3* and *Becn1* were non-significant, their nominal pairwise differences were not interpreted as treatment effects. The significant reduction in *Map1lc3b* mRNA expression with Fetuin-A, together with increased Bcl-2 immunoreactivity, is compatible with modulation of autophagy-related and cell-survival responses, but does not establish altered autophagic flux or a direct anti-apoptotic mechanism. The tissue-protective effects of Fetuin-A show parallels with ischemia/reperfusion models of other organs. In an intestinal ischemia/reperfusion study [18], Fetuin-A administration was reported to suppress autophagic cell death and preserve tissue integrity. In the present ovarian model, the *Map1lc3b* mRNA finding should therefore be regarded as supportive, associative evidence that requires confirmation using protein-level and autophagic-flux assays.

The exploratory STRING analysis further supported functional connectivity among the predefined markers of inflammation, hypoxia, oxidative stress, apoptosis-associated signaling, and autophagy-related responses. The enrichment of oxidative-stress, autophagy, apoptosis, and HIF-1 pathways was biologically consistent with the experimental endpoints evaluated in this study. However, STRING edges represent functional associations derived from multiple evidence sources and do not necessarily indicate direct physical interactions. Moreover, because no expression magnitudes or treatment directions were included in the network analysis, these findings do not demonstrate treatment-induced pathway activation or inhibition, causal mechanisms, or autophagic flux. The isolation of AHSG at the high-confidence threshold should therefore not be interpreted as evidence that Fetuin-A lacks biological relationships with the connected markers.

Clinically, preservation of ovarian reserve is a major objective in the management of adnexal torsion, particularly in reproductive-age and pediatric populations [20]. Ovarian reserve is finite and cannot be replenished; therefore, follicular loss after ischemia/reperfusion injury may have important consequences for future female fertility. In this model, T/D reduced AMH, whereas Fetuin-A pretreatment significantly improved AMH relative to untreated T/D animals. Although AMH alone cannot define long-term fertility potential, its preservation alongside reduced tissue injury and improved cytoprotective marker expression supports a biologically coherent protective effect [5,6]. Pretreatment produced a more consistent protective pattern than administration before detorsion across the measured outcomes; however, the study was not designed or powered for a direct statistical comparison between the two Fetuin-A schedules. Because adnexal torsion is an acute clinical emergency, administration before torsion is not clinically feasible. Nevertheless, the interval between diagnosis and surgical detorsion may permit administration after diagnosis but before reperfusion begins [20]. A systemic or intraperitoneal Fetuin-A regimen delivered during this interval could therefore be evaluated as a clinically relevant adjunctive strategy in future studies.

No clinical studies have evaluated Fetuin-A as a therapeutic intervention for adnexal torsion, and pharmacological strategies aimed at limiting ovarian reperfusion injury remain largely preclinical. In humans, Fetuin-A has primarily been investigated as a circulating biomarker rather than as an administered therapeutic agent. Current management of adnexal torsion relies on prompt surgical detorsion and ovarian-conserving treatment to preserve ovarian function [20]. The present findings are consistent with this clinical objective, as Fetuin-A administration—particularly pretreatment—was associated with preservation of AMH levels and attenuation of inflammatory, hypoxia-related, and histopathological injury. Nevertheless, these results were obtained in a controlled bilateral rat model and should be interpreted as preclinical proof-of-concept evidence rather than evidence of clinical efficacy. Fetuin-A may warrant investigation as a pharmacological adjunct to urgent surgical detorsion, but not as an alternative to surgery. Although administration before torsion is not clinically feasible, treatment after diagnosis and before surgical detorsion could provide drug exposure at the onset of reperfusion. This translational strategy requires validation in unilateral torsion models using clinically feasible post-diagnosis dosing regimens, followed by pharmacokinetic and safety studies and long-term assessment of follicle counts, ovarian function, oocyte quality, pregnancy, and live birth outcomes.

This study has several limitations. First, the sample size was modest, and no a priori power calculation was performed. Consequently, the study may have had limited power to detect modest effects, and the findings should be regarded as exploratory and confirmed in larger, adequately powered experimental cohorts. Second, the bilateral torsion/detorsion model produced reproducible injury and enabled different analyses in the two ovaries, but bilateral torsion is uncommon in clinical practice. A unilateral model with evaluation of both the torsed and contralateral ovary would improve clinical relevance and help distinguish local from systemic effects. Third, inflammatory responses related to surgical manipulation, anesthesia, and the detorsion procedure cannot be completely separated from inflammation caused by ischemia/reperfusion, despite inclusion of the Sham group. Fourth, the study assessed early biochemical, molecular, histological, and immunohistochemical endpoints, but did not evaluate long-term ovarian function, follicle counts, oocyte quality, pregnancy outcomes, or live birth rates. Fifth, protein-level confirmation of the qRT-PCR findings using complementary methods such as Western blotting would strengthen mechanistic interpretation. Finally, only a single Fetuin-A dose and two proof-of-concept administration timings were evaluated; future studies should assess dose–response effects and compare clinically relevant post-diagnosis and post-detorsion schedules using prespecified direct contrasts.

## 4. Materials and Methods

### 4.1. Experimental Animals and Ethical Approval

A total of 28 adult female Wistar albino rats (age: 3–4 months, weight: 250–300 g) were obtained from the Experimental Animals Production, Care, Application and Research Center, Balıkesir University, Balıkesir, Turkey, and included in this study. No a priori power calculation was performed. The sample size of seven animals per group was selected with reference to comparable experimental ovarian torsion/ischaemia–reperfusion studies [3,4,5] and in accordance with the ethical principle of using the minimum number of animals required to obtain interpretable data. The animals were housed under controlled environmental conditions (22 ± 2 °C, 12 h light/dark cycle) with ad libitum access to a standard pellet diet and water. All experimental protocols were approved by the Balıkesir University Local Animal Experiments Ethics Committee (Approval No: 2021/4-13 Date: 20 April 2021) and conducted according to the ARRIVE 2.0 guidelines.

### 4.2. Study Groups and Surgical Procedures

The rats were allocated to four experimental groups (*n* = 7 per group) using a computer-generated randomization sequence: Sham, torsion/detorsion (T/D), T/D+Fetuin-A treatment, and Fetuin-A pretreatment+T/D.

The bilateral 720° torsion model was selected to produce reproducible and sufficiently severe ovarian ischaemia. The 720° rotation is consistent with established rat adnexal torsion models [21], and bilateral torsion with a 3 h ischaemic period has also been described previously [22]. The 2 h reperfusion period was selected on the basis of a rat ovarian ischaemia/reperfusion model demonstrating measurable early biochemical and histopathological injury within this interval [4]. In the present study, bilateral torsion ensured equivalent ischaemia/reperfusion exposure in both ovaries and enabled one ovary from each animal to be used for histopathological and immunohistochemical evaluation and the contralateral ovary for qRT-PCR analysis. Because both ovaries underwent torsion/detorsion, the contralateral ovary could not serve as an untreated internal control.

The 100 mg/kg Fetuin-A dose was selected on the basis of previous in vivo evidence showing dose-dependent protection with systemic Fetuin-A at doses of 20–100 mg/kg [15] and protective effects of repeated 100 mg/kg intraperitoneal Fetuin-A administration in an experimental intestinal ischaemia/reperfusion model [18]. Because the dosing schedules in those studies differed from the present protocol, the two 30 min administration windows were defined a priori as proof-of-concept intervals to compare biologically distinct exposure strategies. Administration before torsion was intended to ensure Fetuin-A availability during both ischaemia and reperfusion, whereas administration before detorsion was intended to ensure availability at the onset of reperfusion, when oxidative and inflammatory injury develops. These timing intervals were experimental choices and should not be interpreted as established or clinically optimized treatment schedules.

1. Sham Group: A dorsal bilateral incision was performed, and the ovaries were gently manipulated without torsion. Animals were sacrificed after 5 h, corresponding to the 3 h torsion plus 2 h reperfusion interval used in the experimental groups, and ovarian tissues were harvested.
2. Torsion/Detorsion Group (T/D): Using a dorsal approach, bilateral adnexa were rotated 720° clockwise for 3 h to induce ischemia. Detorsion was then performed, reperfusion was allowed for 2 h, and ovarian tissues were harvested at sacrifice.
3. T/D+Fetuin-A Treatment Group: The torsion/detorsion protocol was identical to that used in the T/D group. Fetuin-A (Sigma-Aldrich) was dissolved in sterile saline immediately before administration and injected intraperitoneally at a dose of 100 mg/kg 30 min before detorsion.
4. Fetuin-A Pretreatment+T/D Group: Fetuin-A (Sigma-Aldrich) was dissolved in sterile saline immediately before administration and injected intraperitoneally at a dose of 100 mg/kg 30 min before torsion induction, followed by the same torsion/detorsion protocol used in the T/D group.

General anesthesia was induced by intraperitoneal ketamine (80–100 mg/kg) and xylazine (8–10 mg/kg). At the end of the experimental period, animals were euthanized under deep anesthesia by guillotine decapitation, and blood and ovarian tissue samples were collected immediately. After euthanasia, blood samples were collected for serum biochemical analyses. One ovary from each animal was fixed in 10% neutral-buffered formalin for histopathological and immunohistochemical evaluation, while the contralateral ovary was stored at −80 °C for qRT-PCR analysis.

### 4.3. Biochemical Analysis

Blood samples collected at sacrifice were centrifuged, and serum was separated and stored at −80 °C until analysis. Serum interleukin-1β (IL-1β), interleukin-6 (IL-6), and tumor necrosis factor-α (TNF-α) concentrations were measured to evaluate systemic inflammatory activity using commercially available rat ELISA kits: Rat IL-1 beta ELISA Kit (ab255730; Abcam, Cambridge, UK), Rat IL-6 ELISA Kit (ab234570; Abcam), and Rat TNF alpha ELISA Kit (ab46070; Abcam). Serum anti-Müllerian hormone (AMH) was measured as an exploratory endocrine marker related to ovarian reserve using the Rat AMH ELISA Kit (ab323518; Abcam). All assays were performed according to the manufacturer’s instructions.

### 4.4. Quantitative Real-Time PCR (qRT-PCR)

Total RNA was extracted from frozen ovarian tissue stored at −80 °C using Wizol™ Reagent (WISBIOsolution, Seongnam-si, Republic of Korea) according to the manufacturer’s protocol. RNA concentration and purity were assessed spectrophotometrically before complementary DNA (cDNA) synthesis, and samples suitable for downstream analysis were reverse-transcribed using the OneScript^®^ Plus cDNA Synthesis Kit (Applied Biological Materials Inc., Richmond, BC, Canada, Cat. No. G236).

Quantitative real-time PCR was performed using BlasTaq™ 2X qPCR Master Mix (Applied Biological Materials Inc., Cat. Nos. G891 and G892) on a Rotor-Gene Q platform (Qiagen, Düsseldorf, Germany). Reactions were performed in triplicate. The primer sequences used for rat qRT-PCR analysis are provided in Table 1. Expression levels of *Casp3*, *Hmgb1*, *Tlr4*, *Becn1*, and *Map1lc3b* mRNA were normalized to *Actb*, and relative expression was calculated using the 2^−ΔΔCt^ method with Rotor-Gene Q Software version 2.3.4.

### 4.5. STRING Functional Association and Enrichment Analysis

An exploratory functional association analysis was performed using STRING version 12.5 [23] for *Rattus norvegicus* (NCBI taxon 10116). The predefined input panel comprised *Ahsg*, *Hmgb1*, *Tlr4*, *Map1lc3b*, *Becn1*, *Casp3*, *Hif1a*, *Gpx1*, and *Bcl2*. A minimum combined confidence score of 0.700 was applied, all default evidence channels were retained, no additional interactors were added, and disconnected nodes were displayed. Biological Process, KEGG, and Reactome enrichment analyses used the complete nine-protein input and the default rat proteome background. Functional enrichment was considered significant at a STRING-reported Benjamini–Hochberg false discovery rate (FDR) < 0.05. No expression values or treatment-direction data were supplied. MAP1LC3B corresponded to the qRT-PCR target, whereas GPX1 was selected as a representative of the combined GPx-1/2 immunohistochemical readout.

### 4.6. Histopathological and Immunohistochemical Evaluation

After excision, ovarian tissues were fixed in 10% neutral-buffered formalin, processed through routine paraffin-embedding procedures, and cut into 4–5 µm sections. Hematoxylin and eosin (H&E)-stained slides were evaluated semi-quantitatively using predefined 0–3 ordinal scales for inflammation, vascular congestion, and interstitial oedema. For inflammation, 0 indicated no inflammatory-cell infiltration, 1 indicated mild focal infiltration, 2 indicated moderate multifocal infiltration, and 3 indicated marked diffuse infiltration. For vascular congestion, 0 indicated no congestion, 1 indicated mild focal vascular dilatation and erythrocyte accumulation, 2 indicated moderate multifocal congestion, and 3 indicated marked diffuse congestion. For interstitial oedema, 0 indicated no oedema, 1 indicated mild focal stromal separation, 2 indicated moderate multifocal stromal separation, and 3 indicated marked diffuse stromal separation. Representative H&E-stained sections illustrating severe inflammatory-cell infiltration, vascular congestion, and interstitial oedema in untreated torsion/detorsion ovarian tissue are provided in Figure S1.

Immunohistochemical staining was performed on 4–5 µm formalin-fixed, paraffin-embedded (FFPE) ovarian tissue sections. The sections were deparaffinized in xylene three times for 10 min each and rehydrated through a graded series of ethanol solutions (100%, 95%, 80%, and 70%) to distilled water. Antigen retrieval was performed by heating the slides in 10 mM citrate buffer (pH 6.0) in a microwave oven at 95 °C for 15 min, followed by cooling at room temperature for 20 min. Endogenous peroxidase activity was blocked by incubating the sections in a 3% hydrogen peroxide (H_2_O_2_) solution in methanol for 10 min at room temperature.

To prevent non-specific background staining, sections were incubated with a 5% normal goat serum blocking solution for 30 min. The sections were then incubated overnight at 4 °C with the following mouse monoclonal primary antibodies obtained from Santa Cruz Biotechnology: anti-Fetuin-A (H-8, sc-166531, dilution 1:100), anti-HIF-1α (H1alpha 67, sc-53546, dilution 1:50), anti-Bcl-2 (C-2, sc-7382, dilution 1:100), and anti-GPx-1/2 (B-6, sc-133160, dilution 1:100).

To verify the specificity of the antibody labeling and rule out false-positive artifacts, negative control sections were processed simultaneously by omitting the primary antibodies and incubating the tissues exclusively with phosphate-buffered saline (PBS) containing 1% bovine serum albumin (BSA).

Following primary incubation, sections were washed with PBS and incubated with a biotinylated secondary antibody system, followed by the addition of a horseradish peroxidase (HRP)-streptavidin complex for 20 min. Immunoreactivity was visualized using a 3,3′-diaminobenzidine (DAB) chromogen system, and sections were counterstained with Mayer’s hematoxylin, dehydrated through graded alcohols, cleared in xylene, and coverslipped. GPx staining was interpreted as combined GPx-1/2 immunoreactivity.

Immunoreactivity was scored semi-quantitatively according to the percentage of positively stained cells: 0, 0%; 1, 1–33%; 2, 34–66%; and 3, 67–100% positive cells. Thus, the 1+, 2+, and 3+ categories used in the Supplementary Figure captions denote increasing proportions of positively stained cells rather than staining-intensity categories. To minimize assessment bias, all histopathological and immunohistochemical slides were evaluated using an Olympus BX48 light microscope by a single pathologist who was blinded to the treatment groups. The same scoring criteria were applied to all specimens, and no software-based quantitative image analysis was performed.

### 4.7. Statistical Analysis

Statistical analyses were performed using Jamovi software (version 2.3.21). Figures were generated using Python version 3.10 with Matplotlib version 3.11.0. Continuous variables were summarized as mean ± SD. Normality was assessed using the Shapiro–Wilk test, and homogeneity of variance was evaluated before group comparisons. Normally distributed continuous variables were analyzed using one-way ANOVA followed by post hoc pairwise comparisons. When distributional or variance assumptions were not met, Welch’s ANOVA or appropriate non-parametric tests were used. Ordinal histopathological and immunohistochemical scores were analyzed using the Kruskal–Wallis test. When the omnibus test was significant, three pairwise Mann–Whitney U tests compared the Sham, Fetuin-A treatment, and Fetuin-A pretreatment groups separately with the untreated T/D group. The resulting *p*-values were adjusted by the Holm method within each marker to control for multiple comparisons. A two-tailed *p* value <0.05 was considered statistically significant.

## 5. Conclusions

In conclusion, Fetuin-A attenuated early ovarian ischemia/reperfusion injury in this rat adnexal torsion model across systemic, molecular, and tissue-level outcomes. Pretreatment produced the most consistent benefit, reducing serum TNF-α levels, attenuating *Hmgb1*, *Tlr4*, and *Map1lc3b* mRNA expression, improving histopathological injury scores, increasing GPx and Bcl-2 immunoreactivity, reducing HIF-1α immunoreactivity, and preserving AMH levels relative to untreated torsion/detorsion. These findings support an association between Fetuin-A administration and reduced inflammatory, hypoxia-related, and cell-stress responses but do not establish direct pathway causality, altered autophagic flux, or long-term preservation of fertility. Further studies are warranted to define optimal dosing, treatment timing, mechanistic pathways, and long-term reproductive outcomes before clinical translation.

## Figures and Tables

**Table 1:** Primer sequences used for rat qRT-PCR analysis.

Target Gene	Forward Primer (5′–3′)	Reverse Primer (5′–3′)
*Actb*	CCCGCGAGTACAACCTTCT	CGTCATCCATGGCGAACT
*Casp3*	GGTATTGAGACAGACAGTGG	CATGGGATCTGTTTCTTTGC
*Hmgb1*	GAGTACCGCCCAAAAATCAA	TTCATCCTCCTCGTCGTCTT
*Tlr4*	AATTCCTCAGACGGTGGCAG	CCGAGCAGGTTAGATGACAA
*Becn1*	ATGGAGGGGTCTAAGGCGTC	TCCTCTCCTGAGTTAGCCT
*Map1lc3b*	CGCACCTTCGAACAGATGAT	CCAACCCGTATGCTTGGAT

## Data Availability

The data presented in this study are available on reasonable request from the corresponding author.

## Supplementary Materials

The following supporting information can be downloaded at: https://www.mdpi.com/article/10.3390/biomedicines14102282/s1, Figure S1. Representative H&E-stained sections showing histopathological changes in ovarian tissue from the torsion/detorsion group. (Left) Severe inflammatory cell infiltration in the ovarian stroma with dilated congested vessels and interstitial oedema (×100). (Middle) Severe vascular congestion in the ovarian stroma (×100). (Right) Severe interstitial oedema in the ovarian stroma (×100); Figure S2. Representative immunohistochemical staining of HIF-1α in the ovarian stroma. (Left) Moderate cytoplasmic HIF-1α immunoreactivity (2+) in the Fetuin-A treatment group (×400). (Right) Strong cytoplasmic HIF-1α immunoreactivity (3+) in the untreated torsion/detorsion group (×200); Figure S3. Representative immunohistochemical staining of GPx in the ovarian stroma. (Left) Mild cytoplasmic GPx immunoreactivity (1+) in the Sham group (×400). (Middle) Moderate cytoplasmic GPx immunoreactivity (2+) in the Fetuin-A pretreatment group (×400). (Right) Strong cytoplasmic GPx immunoreactivity (3+) in the Fetuin-A pretreatment group (×200); Figure S4. Representative immunohistochemical staining of Bcl-2 in the ovarian stroma. (Left) Moderate cytoplasmic Bcl-2 immunoreactivity (2+) in the Fetuin-A pretreatment group (×200). (Right) Strong cytoplasmic Bcl-2 immunoreactivity (3+) in the Fetuin-A pretreatment group (×200); Figure S5. Representative immunohistochemical staining of Fetuin-A in the ovarian stroma. (Left) Mild cytoplasmic Fetuin-A immunoreactivity (1+) in the Sham group (×400). (Middle) Moderate cytoplasmic Fetuin-A immunoreactivity (2+) in the Fetuin-A pretreatment group (×400). (Right) Strong cytoplasmic Fetuin-A immunoreactivity (3+) in the Fetuin-A pretreatment group (×200); Figure S6. STRING functional association network of the selected rat protein panel comprising AHSG, HMGB1, TLR4, MAP1LC3B, BECN1, CASP3, HIF1A, GPX1, and BCL2. STRING version 12.5 was queried for *Rattus norvegicus* using a minimum combined confidence score of 0.700, all default evidence sources, and no additional interactors. Edge thickness indicates association confidence. Disconnected nodes were retained; AHSG had no qualifying association with another input protein at this threshold. The network contained 9 nodes and 17 associations, compared with 1 expected association (PPI enrichment *p* = 5.11 × 10^−15^). Edges indicate functional associations and do not necessarily represent direct physical binding; Table S1. Selected significant STRING functional enrichment terms relevant to the predefined nine-protein rat panel. FDR values were calculated using the Benjamini–Hochberg correction.

## Abbreviations

FDR, false discovery rate; GO, Gene Ontology; KEGG, Kyoto Encyclopedia of Genes and Genomes. Input count indicates the number of proteins from the predefined nine-protein panel assigned to each term; background count indicates the number of proteins assigned to that term in the default rat proteome. Raw *p*-value denotes the unadjusted enrichment probability, whereas FDR denotes the Benjamini–Hochberg-adjusted value. Only study-relevant terms with FDR < 0.05 are shown.
