# Supplementary material for: Fetuin-A Attenuates HMGB1/TLR4-Mediated Ovarian Ischemia/Reperfusion Injury
Source: Biomedicines. 2026 Oct 8;14(10):2282. doi: 10.3390/biomedicines14102282 (PMC13701938; doi:10.3390/biomedicines14102282)
Supplement: Supplementary file 1 [file biomedicines-14-02282-s001.zip › biomedicines-4580448-supplementary.pdf]

Supplementary Materials:

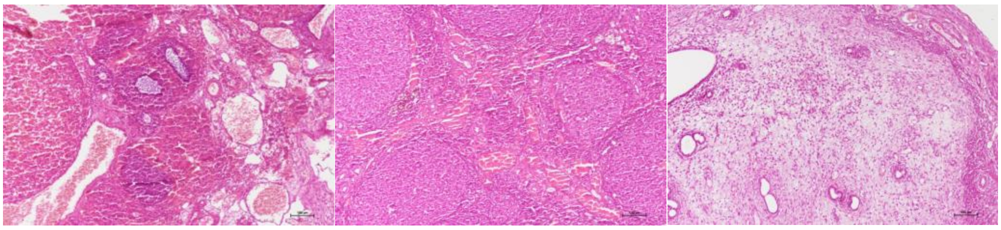

Figure S1. Representative H&E-stained sections showing histopathological changes in ovarian tissue from the torsion/detorsion group. (Left) Severe inflammatory cell infiltration in the ovarian stroma with dilated congested vessels and interstitial oedema (×100). (Middle) Severe vascular congestion in the ovarian stroma (×100). (Right) Severe interstitial oedema in the ovarian stroma (×100).

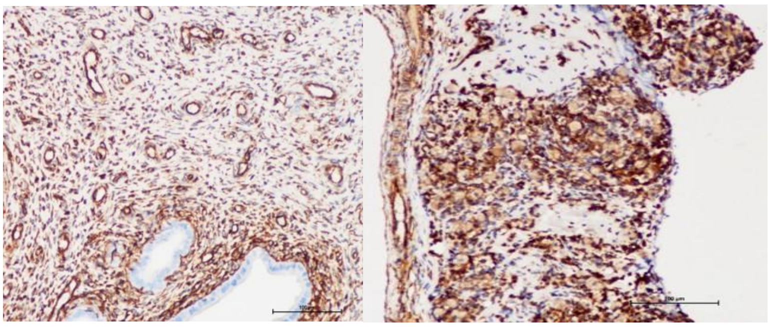

Figure S2. Representative immunohistochemical staining of HIF-1α in the ovarian stroma. (Left) Moderate cytoplasmic HIF-1α immunoreactivity (2+) in the Fetuin-A treatment group (×400). (Right) Strong cytoplasmic HIF-1α immunoreactivity (3+) in the untreated torsion/detorsion group (×200).

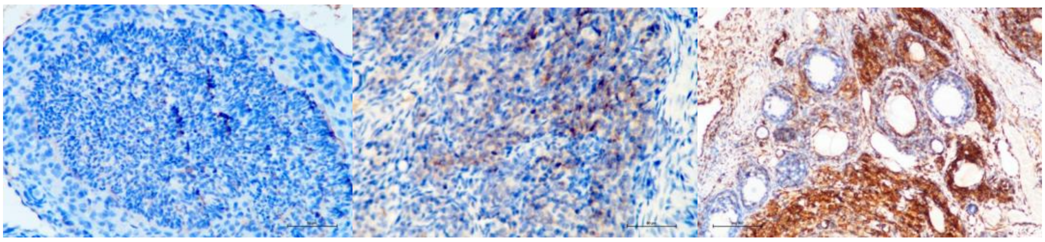

Figure S3. Representative immunohistochemical staining of GPx in the ovarian stroma. (Left) Mild cytoplasmic GPx immunoreactivity (1+) in the Sham group (×400). (Middle) Moderate cytoplasmic GPx immunoreactivity (2+) in the Fetuin-A pretreatment group (×400). (Right) Strong cytoplasmic GPx immunoreactivity (3+) in the Fetuin-A pretreatment group (×200).

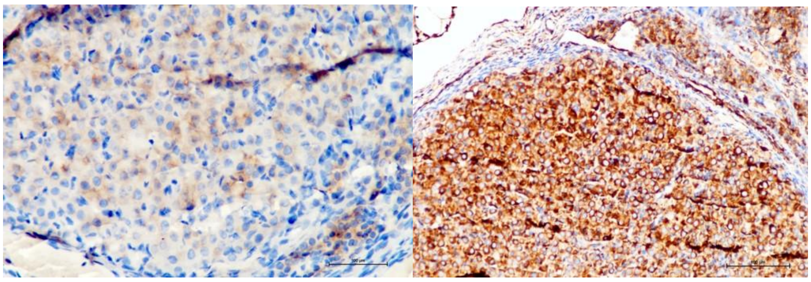

Figure S4. Representative immunohistochemical staining of Bcl-2 in the ovarian stroma. (Left) Moderate cytoplasmic Bcl-2 immunoreactivity (2+) in the Fetuin-A pretreatment group (×200). (Right) Strong cytoplasmic Bcl-2 immunoreactivity (3+) in the Fetuin-A pretreatment group (×200).

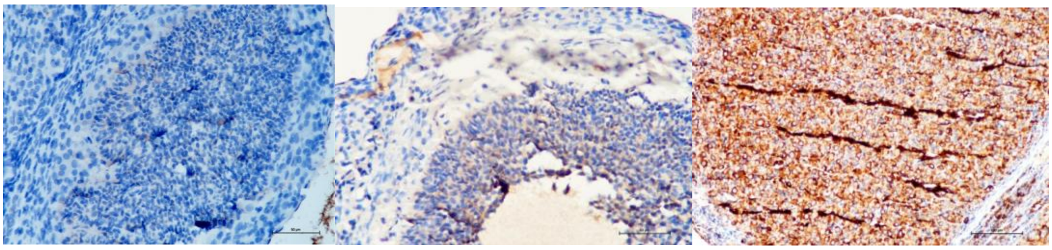

Figure S5. Representative immunohistochemical staining of Fetuin-A in the ovarian stroma. (Left) Mild cytoplasmic Fetuin-A immunoreactivity (1+) in the Sham group (×400). (Middle) Moderate cytoplasmic Fetuin-A immunoreactivity (2+) in the Fetuin-A pretreatment group (×400). (Right) Strong cytoplasmic Fetuin-A immunoreactivity (3+) in the Fetuin-A pretreatment group (×200).

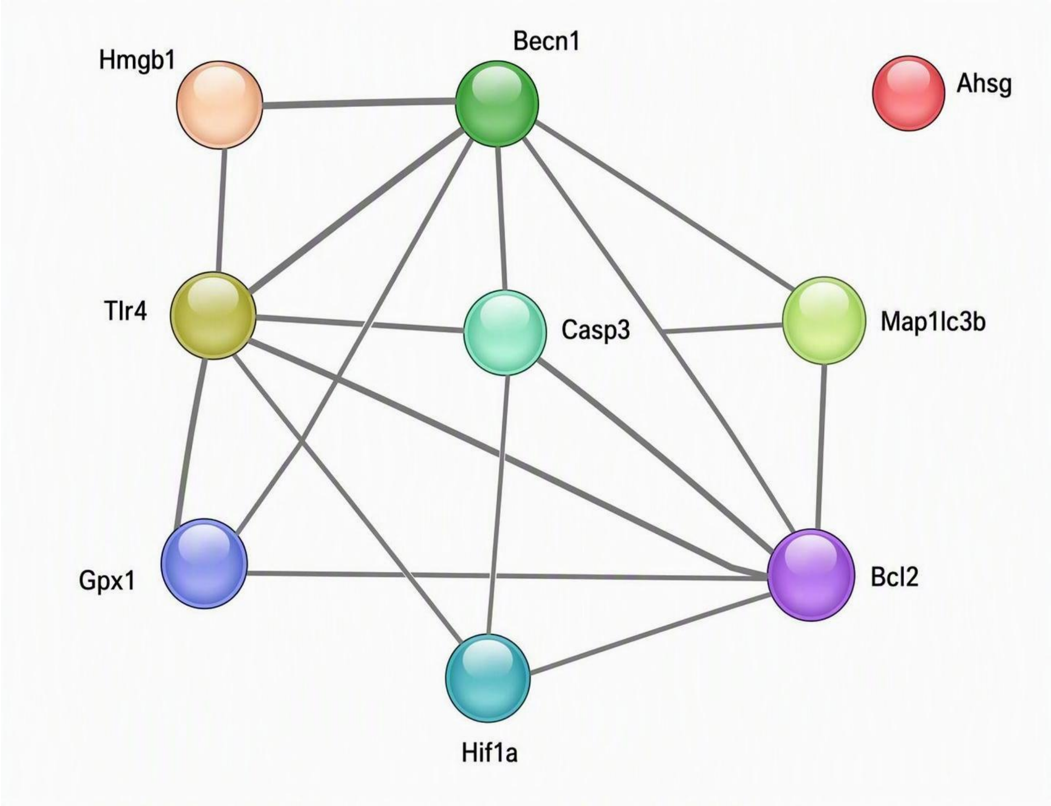

Figure S6. STRING functional association network of the selected rat protein panel comprising AHSG, HMGB1, TLR4, MAP1LC3B, BECN1, CASP3, HIF1A, GPX1, and BCL2. STRING version 12.5 was queried for *Rattus norvegicus* using a minimum combined confidence score of 0.700, all default evidence sources, and no additional interactors. Edge thickness indicates association confidence. Disconnected nodes were retained; AHSG had no qualifying association with another input protein at this threshold. The network contained 9 nodes and 17 associations, compared with 1 expected association (PPI enrichment $p = 5.11 \times 10^{-15}$). Edges indicate functional associations and do not necessarily represent direct physical binding.

Table S1. Selected significant STRING functional enrichment terms relevant to the predefined nine-protein rat panel. FDR values were calculated using the Benjamini–Hochberg correction.

Category	Term ID	Term description	Input count	Background count	Raw p-value	FDR	Contributing proteins
Biological Process	GO:0006979	Response to oxidative stress	6	457	6.27×10^{-9}	4.28×10^{-5}	BCL2; TLR4; CASP3; HIF1A; GPX1; BECN1
KEGG	rno04140	Autophagy – animal	4	259	2.30×10^{-6}	4.40×10^{-4}	BCL2; HIF1A; HMGB1; BECN1
KEGG	rno04215	Apoptosis – multiple species	3	42	6.44×10^{-7}	2.40×10^{-4}	BCL2; CASP3; BECN1
KEGG	rno04066	HIF-1 signaling pathway	3	158	3.03×10^{-5}	0.0023	BCL2; TLR4; HIF1A

Abbreviations: FDR, false discovery rate; GO, Gene Ontology; KEGG, Kyoto Encyclopedia of Genes and Genomes. Input count indicates the number of proteins from the predefined nine-protein panel assigned to each term; background count indicates the number of proteins assigned to that term in the default rat proteome. Raw p-value denotes the unadjusted enrichment probability, whereas FDR denotes the Benjamini–Hochberg-adjusted value. Only study-relevant terms with FDR < 0.05 are shown.
